# Is dual testing for hepatitis C necessary? Modelling the risk of removing hepatitis C antibody testing for Australian blood donations

**DOI:** 10.1111/vox.13430

**Published:** 2023-05-14

**Authors:** Avijoy Roy Choudhury, Veronica C. Hoad, Clive Seed, Peter Bentley

**Affiliations:** ^1^ UWA Medical School The University of Western Australia Perth Western Australia Australia; ^2^ Australian Red Cross Lifeblood Perth Western Australia Australia

**Keywords:** blood donation testing, blood safety, hepatitis C virus, transfusion‐transmissible infections

## Abstract

**Background and Objectives:**

Parallel testing of blood donations for hepatitis C virus (HCV) antibody and HCV RNA by nucleic acid testing (NAT) has been standard practice in Australia since 2000. Meanwhile, NAT technologies have improved, and HCV has become a curable disease. This has resulted in a significant reduction in the risk and clinical consequences of HCV transmission through transfusion. This study aimed to estimate the residual risk (RR) under various testing options to determine the optimal testing strategy.

**Materials and Methods:**

A developed deterministic model calculated the RR of HCV transmission for four testing strategies. A low, mid and high estimate of the RR was calculated for each. The testing strategies modelled were as follows: universal dual testing, targeted dual testing for higher risk groups (first‐time donors or transfusible component donations) and universal NAT only.

**Results:**

The mid estimate of the RR was 1 in 151 million for universal dual testing, 1 in 111 million for targeted dual testing of first‐time donors, 1 in 151 million for targeted dual testing for transfusible component donations and 1 in 66 million for universal NAT only. For all testing strategies, all estimates were considerably less than 1 in 1 million.

**Conclusion:**

Antibody testing in addition to NAT does not materially change the risk profile. Even conservative estimates for the cessation of anti‐HCV predict an HCV transmission risk substantially below 1 in 1 million. Therefore, given that it is not contributing to blood safety in Australia but consuming resources, anti‐HCV testing can safely be discontinued.

**Highlights**

- Parallel testing of blood donations for hepatitis C virus (HCV) antibody and HCV RNA by nucleic acid testing (NAT) has been standard practice in many jurisdictions since 2000.
- Testing technology refinements as well as curative treatment for HCV has changed the transfusion‐transmission risk paradigm in Australia.
- Using a deterministic model to estimate residual risk (RR) for several HCV testing strategies, we conclude that if HCV RNA ID‐NAT is performed, anti‐HCV testing is no longer required to maintain a tolerable RR.

## INTRODUCTION

Since the discovery of the hepatitis C virus (HCV), the risk of transfusion‐transmitted HCV has decreased substantially. Whilst immunoassay development [1] and subsequent improvement in testing resulted in substantial reductions in [2] the risk of anti‐HCV testing, it remained limited by the 7–8‐week antibody testing window period [3]. Nucleic acid testing (NAT) targeting HCV RNA developed in the late 1990s significantly reduced the residual risk (RR) [4].

The current testing strategy for HCV in blood donations for transfusible components in Australia involves performing both anti‐HCV testing and individual donor‐nucleic acid testing (ID)‐NAT. Plasma for further manufacture donations are currently tested for anti‐HCV and NAT in 16 donation pools (MP‐16). The HCV context has substantially changed. The development of direct‐acting anti‐viral drugs (DAAs) has made chronic HCV a curable disease [5]. Anti‐HCV testing does not differentiate between the decreasing small number of donors at risk of transmitting with active infection and resolved infection.

The Alliance of Blood Operators' Risk‐Based Decision‐Making Framework determines transmission risk and cost effectiveness as fundamentals in blood safety decision‐making [6, 7]. Proposed interventions should be assessed for their likelihood of mitigating the risk and the proportional resource allocation in comparison with similar risks to the blood system or health system [8]. Australian Red Cross Lifeblood's (Lifeblood) risk tolerability framework defines the tolerable risk for HCV transmission as less than 1 in 1 million (Lifeblood document), which considers other important factors in the risk assessment process including reputational risk, stakeholder assessment and societal viewpoints.

An international NAT study assessing over 10 million donations parallelly tested with NAT and anti‐HCV concluded that anti‐HCV testing was adding very little additional risk reduction [9]. Lifeblood remains committed to providing both a safe and cost‐justified service to the Australian public, which has prompted consideration of alternative screening testing strategies in the changing HCV context.

This study models the risk of transfusion‐transmitted HCV in four alternative testing strategies, enabling a subsequent economic analysis of cost effectiveness [10] to determine the optimal HCV testing strategy.

## MATERIALS AND METHODS

A simple deterministic risk model was developed under various testing strategies with a risk evaluation model considering the RR as calculated and other risks, including an evaluation of risk tolerability with any testing failure and recipient impacts.

### Selection of targeted‐testing strategies

Consideration of various potential testing strategies was made based on Australian donation patterns and the known HCV donor prevalence. Lifeblood is plasma collection focussed with approximately 55% of all 2020 donations being apheresis plasma. The rate of HCV‐positive donations (defined as being either anti‐HCV‐positive and/or NAT‐positive) was 51.5 and 0.67 per 100,000 donations in new and repeat donors, respectively [11]. First‐time donor donations were categorized as a higher testing positive risk. While this is a cumulative prevalence and does not translate to the equivalent impact on transmission, this impacts on the prevalence of detection of anti‐HCV in blood donations, considered separately in the risk assessment.

Transfusible component donations were categorized as higher risk compared to plasma for further manufacture because of dedicated viral inactivation and removal processes during fractionation [12], which are effective against enveloped viruses such as HCV [13].

Therefore, the status quo was compared to three alternative testing strategies (Table 1). For each testing strategy, the RR was calculated as either a low (likely most representative), mid (midpoint between low and high) or high (worst case) estimate based on varying assumptions expanded on below.

**TABLE 1:** Proposed hepatitis C testing strategies for blood donation.

Testing strategy 1	Universal testing with both NAT and anti‐HCV (current testing strategy in Australia)
Testing strategy 2	Targeted testing with NAT and anti‐HCV testing for first‐time donors and NAT for remaining donations
Testing strategy 3	Targeted testing with ID‐NAT and anti‐HCV for transfusible components and MP‐16 NAT for plasma for further manufacture
Testing strategy 4	Universal donation NAT only (complete removal of anti‐HCV testing)

*Note*: Nucleic acid testing (NAT) is for individual donor for transfusible component donations and MP‐16 for plasma for further manufacture.

Abbreviation: HCV, hepatitis C virus.

### Testing strategy 1 (status quo: ID anti‐HCV testing for all donations, ID‐NAT for transfusible components, MP‐NAT for plasma for further manufacture)

Testing strategy 1 is the baseline current testing strategy. The RR for this testing strategy was derived using Lifeblood HCV donation testing data for the 6‐year period (2015–2020) using the model established by Weusten et al. [14], which is calculated routinely by Lifeblood (see Supporting information).

### Testing strategies 2 and 4

The RR for testing strategies 2 and 4 was calculated by adjusting the baseline RR by adding the estimated RR increase if anti‐HCV was removed from the selected population in the respective testing strategies. The additional risk was calculated by the product of the following parameters:

1. Prevalence of anti‐HCV‐positive, NAT‐non‐reactive donations in the population if anti‐HCV testing is no longer performed in either repeat donors (strategy 2) or removed completely (strategy 4) (internal data).

Note that if anti‐HCV testing is withdrawn, anti‐HCV‐positive donors would no longer be detected and therefore the true, but now undetected anti‐HCV prevalence in the donors would increase. Importantly, this does not constitute a change in the HCV incidence, but rather non‐detection of past infections. The purpose of the model is to estimate potentially infectious anti‐HCV‐positive ID‐NAT non‐reactive donations. The rate of these would not be expected to further change in our model, given that non‐detectable viraemia in anti‐HCV‐positive donations is assumed to occur after seroconversion during clearance of viraemia (i.e., during a defined period during viral clearance), and, unlike in occult hepatitis B infection, is not expected to occur intermittently given that occult HCV is defined by the absence of RNA detection in the serum [15].

The prevalence of anti‐HCV‐positive, NAT‐non‐reactive donations was derived from the 2016 to 2020 Lifeblood donation testing data.

2 The percentage of potentially infectious anti‐HCV‐positive, NAT‐non‐reactive donations.

In brief, El Ekiaby et al. [16], in the previous highest world‐wide estimated HCV prevalence country, aimed to determine the prevalence of low‐level viraemia in donors with a testing pattern consistent with resolved HCV infection (i.e., antibody‐positive, NAT‐negative donations). This is dependent on the incidence, given that this event is postulated to occur in the period during which infectivity is resolving and there will be a period, similar to the acute window period, where the virus will be present but undetectable by NAT. In this study, 175 resolved samples were evaluated for presence of low‐level vireamia by replicate HCV‐RNA testing (*n* = 10) in the Grifols Ultrio NAT. In the Egyptian population, 2 of 174 (0.114%) were positive. This estimate was used as our ‘high estimate’, but given that the incidence of HCV in Egypt as demonstrated by the same studies' NAT yield results was 1 in 7145 donations and total first‐time donor HCV prevalence was 2.86%, this is a beyond ‘worst case’ Australian estimate. An adjustment factor for the Australian population was derived by comparing the anti‐HCV‐positive prevalence in Australian first‐time donors (107 positive of 501,450 donations) compared to Egyptian donors (937 antibody‐positive only per 119,756 donations), which was 36.7 times lower.

3 The percentage of assumed infectious anti‐HCV‐positive but NAT‐negative donations resulting in a recipient infection (transmission factor).

The transmission factor was from El Ekiaby et al. [16]. Using Poisson distribution formulas [17] and a minimum infectious dose of 316 virions for anti‐HCV‐reactive transfusions [18], the authors estimated that the two low viraemic donations (which contained 0.5 and 1.8 copies/mL HCV‐RNA, respectively) had probabilities of 1.1% and 3.9% to be infectious after transfusion of a red blood cell unit containing 20 mL of plasma and 10.4% and 32.6% for transfusion of a 200‐mL fresh frozen plasma (FFP) unit. Therefore, the transmission factor for infectious donations used was 0.025 for red cells and 0.215 for clinical plasma. Australian 2020 donation data were used to determine the proportion of red cells and plasma. Given that Lifeblood platelets are either suspended in a platelet additive solution or a small amount of plasma, platelets were given the same transmission factor as red cells. In 2020, 76.3% of Lifeblood's components were red cells or platelets and 23.7% clinical plasma. It is noted that Lifeblood has a significant cryoprecipitate inventory, so the mean volume of clinical plasma is less than the modelled risk.

An overview of the calculation methodology is presented in Table 2. The proportional increase for each strategy was added to the original RR.

**TABLE 2:** Strategy 2 and 4 risk adjustment methodology.

Risk adjustment methodology	Data source
Prevalence of anti‐HCV‐positive, ID‐NAT‐non‐reactive donations in the donor population if anti‐HCV testing is no longer performed (internal data)	See Table 3
Proportion anti‐HCV infectious [16]	0.114%
Adjustment factor (proportion first‐time donors anti‐HCV positive: Egypt to Australia)	36.7 times lower High estimate: No adjustment factor Low estimate: Full adjustment factor Mid estimate: Half adjustment factor
Infectious donation transmission risk [16]	Red cells: 0.025 Clinical plasma: 0.215
Proportion donations clinical plasma Proportion red cell or equivalent (Lifeblood internal data)	0.237 0.763

Abbreviations: HCV, hepatitis C virus; ID‐NAT, individual donor‐nucleic acid testing.

### Testing strategy 3

Plasma for further manufacture incorporates viral reduction/removal processes. The assumption was that any additional low NAT‐positive donation undetected by MP‐NAT would not materially alter the RR, given the low incidence. Therefore, the potential for a positive donation below the level of detection undergoing fractionation was considered when determining the RR for testing strategy 3, and how much this would increase if not testing anti‐HCV donations.

A classical incidence‐window period deterministic model was used to calculate the baseline RR with testing adjusted for pools of 16 (MP‐16) (see Data S1). The additional risk of an anti‐HCV infectious donation was added by determining the prevalence of anti‐HCV untested donations based on the number of anti‐HCV‐positive donations of plasma (see Table 3), assuming that 0.114% were RNA positive but not detected [16], with no adjustment for the Australian population or minipool testing. These two risk figures were then added together to determine the estimated number of potentially infectious but NAT‐negative donations for a total risk.

**TABLE 3:** Number of Lifeblood blood donations that tested positive using anti‐hepatitis C virus (HCV), HCV nucleic acid testing, or both, between 2016 and 2020.

Donation categories	Anti‐HCV‐positive, NAT reactive	Anti‐HCV‐positive, NAT non‐reactive	Anti‐HCV‐negative, NAT reactive (NAT yield)
First‐time donations (*n* = 501,450)	101	107	0
Repeat donations (*n* = 6,605,800)	17	41	1
Transfusible component donations (*n* = 3,845,771)	105	133	1
Plasma for further manufacture (*n* = 3,261,479)	13	15	0
Total	118	148	1

Abbreviation: NAT, nucleic acid testing.

The risk of an infectious donation below the level of detection in MP‐16 (48 IU/mL) was then used to calculate the maximum viral load in an 850‐mL bag of plasma. A log reduction with factor of 10.6 [19] was then applied to calculate the maximum virion load in an 850‐mL bag following fractionation to determine transmission risk.

### Other risks to recipients

**FIGURE 1:**
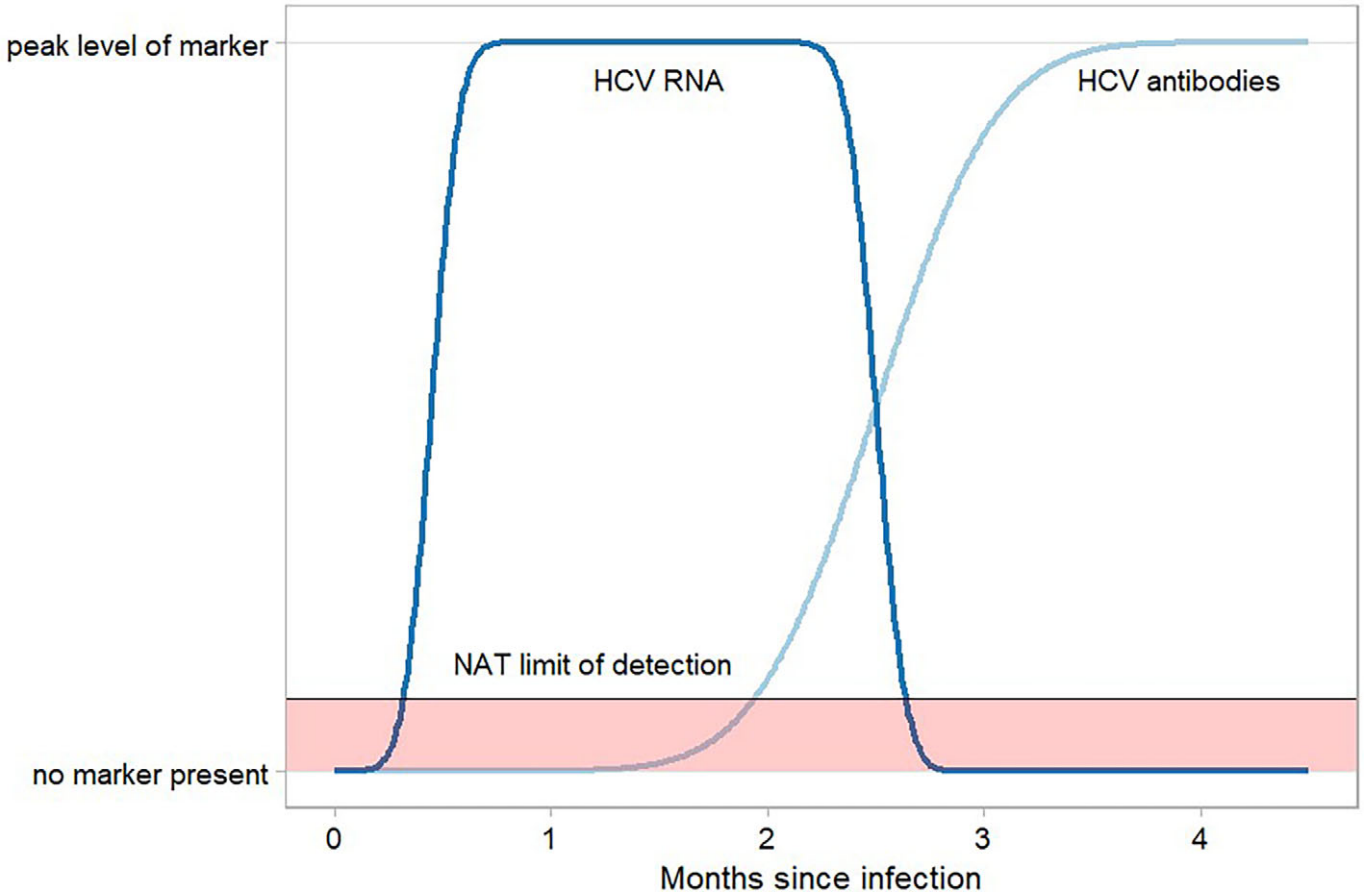
Schema of periods where nucleic acid testing can be false negative as below the NAT limit of detection following infection and ramp‐up (window period, where HCV antibodies are not detectable) and during resolving infection where HCV antibodies are detectable. NAT, nucleic acid testing; HCV, hepatitis C virus (figure by Claire Styles).

The assumption in the model is that anti‐HCV‐positive/NAT‐negative donations are infectious only for a limited period (see Figure 1) and a single donor does not constitute an ongoing transmission risk. This assumption is based on evidence that a single test at 12 weeks after treatment is adequate to be considered cured [20] and a high concordance of 12‐week results [21]. Australian donation guidelines have a 2‐week donation interval, and very few donors donate at the minimum interval. However, there is a risk of passive transfer of anti‐HCV to a recipient. Prior to widespread anti‐HCV screening of blood donors, high concentrations in immunoglobulin preparations were reported, prompting a recommendation to implement plasma donor anti‐HCV screening to prevent passive transfer from interfering in patient monitoring [22]. This could result in test misinterpretation, predominately in recipients of plasma donations, and this is mitigated by removing anti‐HCV‐positive donations. The risk of transfusing an anti‐HCV‐positive donation with the capacity to interfere with recipient test results was calculated, considering the prevalence would be the first‐time donor prevalence, if testing was discontinued.

### Risk of a process failure

Continuing anti‐HCV testing provides redundancy in case of a process failure of NAT, where both tests have to fail for transfusion‐transmission. To demonstrate that this is largely a theoretical risk, a process failure model was developed with the current anti‐HCV‐positive RNA‐positive donations for testing strategies 2 and 4. The rate of positive RNA donations was determined from internal data for first‐time and repeat donors. For *no testing*, the rate of RNA‐positive donations was the total rate in first‐time and repeat donors, and for *first‐time donor testing*, the rate was the rate in repeat donors. For plasma for fractionation, the model accounted for MP‐16 (the risk increased by 16).

$$\begin{array}{l} \text{Process failure rate additional risk}\\ \;=\begin{array}{l} \text{Various risks of}\;a\;\text{process failure}\times \text{risk of an}\;\mathrm{RNA}\\ -\;\text{positive donation that does not have dual testing under the strategies.} \end{array} \end{array}$$

## RESULTS

### Screening data

Over the 2016–2020 period, 7,107,210 donations were included. Of these, 118 donations tested positive for HCV by both NAT and antibody testing, 148 anti‐HCV positive only and 1 NAT yield (see Table 3).

### Residual risks

Table 4 presents the four testing strategies' HCV transmission RRs.

**TABLE 4:** Estimated residual risk of hepatitis C virus transmission for each testing strategy.

	Low estimate	Mid estimate	High estimate
Testing strategy 1	1 in 151 million	1 in 151 million	1 in 151 million
Testing strategy 2	1 in 148 million	1 in 111 million	1 in 89 million
Testing strategy 3	1 in 151 million	1 in 151 million	1 in 151 million
Testing strategy 4	1 in 146 million	1 in 66 million	1 in 43 million

The RR in a plasma for further manufacture was estimated to introduce an additional 0.17 extra donations below the threshold of NAT but positive in the 5‐year period, with a risk of a NAT‐negative donation containing infectious virions being 1 in 16.4 million. The estimated number of virions left after fractionation with a worse case unit was 2.77 × 10^−6^. This number is substantially less than the postulated 50% minimum infectious dose of 7–20 copies [18]. Therefore, we conclude that the RR in a plasma for further manufacture is no greater than the baseline risk and the RR is unchanged.

### Passive transfer risk

If anti‐HCV antibody testing was discontinued, the rate of donations testing positive could be expected to stabilize at the first‐time donor rate of approximately 1 in 4700.

### Process failure risk

The process failure risks are outlined in Table 5. For each strategy of no testing, first‐time donor testing and transfusible component testing, a process failure would need to occur, on average 1 in every 16.7, 2.6 and 103 donations, respectively, to increase release of a viraemic unit to more than 1 in 1 million.

**TABLE 5:** Event of a process failure and impact.

Process failure rate of the NAT (1 in *x*)	Testing strategy 4: Additional risk (1 in *x*)	Testing strategy 2: Additional risk (1 in *x*)	Testing strategy 3: Additional risk of contaminated fractionated plasma unit (1 in *x*)
100,000	5,972,478,992	38,857,647,059	962,481,250
10,000	597,247,899	3,885,764,706	96,248,125
1000	59,724,790	388,576,471	9,624,812
100	5,972,479	38,857,647	962,481
10	597,248	3,885,765	96,248
2	119,450	777,153	19,249

Abbreviation: NAT, nucleic acid testing.

## DISCUSSION

Our modelling demonstrates that anti‐HCV testing is not required to maintain a tolerable transfusion‐transmission HCV risk, even using our unrealistically high estimate. Therefore, changing to a testing strategy that is more cost effective should be considered, and accordingly the risks derived here have been applied to a separate cost‐effectiveness analysis [10].

Cappy et al. [23] estimated that 0.5% of anti‐HCV NAT‐negative (including a period of pooled testing) had low‐level RNA. This value is lower than our high estimate. A formal RR was not performed in this work and, importantly, our argument is about cost effectiveness [10] for a test that does not materially change the RR.

Our findings of dual testing inefficiency are not novel. A large, multi‐country NAT study published in 2015 demonstrated that the efficacy of HCV NAT in removing HCV transmission risk per unit of blood was 99.98% in first‐time donors and 97.94% in repeat donors [9]. The authors concluded that the efficacy increase of anti‐HCV testing when ID‐NAT screening is performed was minimal.

The Australian overall notification rate of HCV declined by 31% over a 10‐year period, as has the proportion of potentially infectious donors (i.e., RNA‐positive cases) [11]. Australia provides free treatment for HCV with DAAs [24], resulting in cure in over 95% [5]. Not only has this contributed to a decreasing transfusion‐transmitted RR because of declining HCV incidence, but it has also lessened morbidity/mortality. These developments favour transitioning to a more cost‐effective HCV donation testing strategy.

Performing two HCV screening tests to address a potential process failure associated with a single test process is one argument to maintain anti‐HCV testing. However, when we modelled the process failure rates and associated RRs by strategy, it was clear that very high failure rates in a single test (NAT) were required to result in an intolerable RR. To breach Lifeblood's 1 in 1 million intolerable threshold, a process failure for NAT would need to occur on average 1 in every 2.6–16.7 donations. While the exact Lifeblood failure rate of NAT is unknown, the demonstrated failure rates required to impact materially are clearly outside the bounds of how often a process failure would occur with good laboratory practices and is therefore a negligible risk.

Blood donation testing for traditional transfusion‐transmitted infections  evolved over time in Australia with the addition of more sensitive tests [25]. However, single‐test serological strategies were used effectively in Australia prior to NAT implementation [26, 27]. West Nile virus single testing, which uses NAT [28], provides adequate protection against viraemia that may be as high as 1 in 1057. The exemplary safety profile of ID‐NAT for HCV is supported by the absence of any ID‐NAT reported cases of transfusion‐transmission. In addition, in the resolving phase of infection with viraemia below the level of detection, there is decreased infectivity [18] compared to the same viral load in the ramp‐up phase, which is thought to be due to viral particle immune complexes and neutralizing antibodies.

Although complete cessation of anti‐HCV testing has operational advantages and is the most cost‐effective [10], there are reasons why first‐time donor testing may be regarded as the optimal initial change. Given that first‐time donors are only 11.5% of total Lifeblood tests (and decreasing over time) and account for 73% of all anti‐HCV positives, anti‐HCV testing costs could be reduced by approximately 90%. First‐time donor screening would also prevent the majority of true‐positive anti‐HCV donors with cleared infection entering the blood donor pool, which would avoid potential downstream issues, such as passive transfer. Passive transfer could theoretically cause transient false positivity in recipients receiving FFP and concentrated plasma products if tested in the months post transfusion. However, given that this is a measure of past exposure, this would not impact on a treatment plan. If first‐time anti‐HCV‐positive donors were tested and removed from the donor pool, this risk would be sufficiently rare to be a negligible for transfusible components, given the dilution factor. In Australia, we have recently ceased serological screening for both human T‐cell lymphotropic virus and syphilis in plasma donations for further manufacture, excluding first‐time donor testing, because of a negligible transmission risk. This is despite the potential for passive transfer, demonstrating that this consequence alone is not a reason to continue testing in the absence of a transmission risk.

False‐positive results are a significant issue in testing blood donations [29]. False positivity is a random event, and therefore eliminating the majority of testing (repeat donors) will eliminate much of the issue of false‐positive results. This would reduce the operational load of complex and time‐consuming counselling as well as costly discard of non‐issuable false‐positive donations.

We considered anti‐HCV cessation and the potential risk of occult HCV. Occult HCV is defined as the presence of HCV RNA in hepatocytes or peripheral blood mononuclear cells (PBMCs) while being absent in the serum [30]. Considering NAT measures HCV RNA in the serum, it is unable to detect occult HCV infections. No Australian cases of occult HCV have been reported. Studies estimate from 0.15% to 85% of being able to detect RNA, depending on the population group. However, all testing involves PBMCs or hepatocytes [30] and this is not synonymous with a transfusion‐transmission risk, as demonstrated with other viruses such as EBV, which, while detectable lifelong in lymphocytes, do not represent a chronic transmission risk [31]. We consider that occult HCV remains only a theoretical transfusion‐transmission risk.

Anti‐HCV testing in addition to multiplex NAT (which simultaneously mitigates HIV, HBV and HCV risk) does not contribute to blood safety in Australia, while adding substantial cost. In keeping with risk‐based decision‐making principles and blood operators moving away from preventing extremely rare risks at any cost, as evidenced by the recent argument in favour of continuation with MP NAT in Germany despite an extremely rare HCV minipool transmission [32], our risk tolerability threshold incorporates societal expectations and appropriate resource use. Given that the marginal risk reduction does not materially change the RR for transfusion recipients, our modelling demonstrates that, even using conservative assumptions, this is an ineffectual use of resources. Complete cessation of anti‐HCV testing is operationally the simplest option. Based on our findings, we intend to progress an application to our regulator to cease anti‐HCV testing.

## CONFLICT OF INTEREST STATEMENT

The authors declare no conflicts of interest.

## Supporting information


**Data S1.** Supporting information
