# Supplementary material for: Is dual testing for hepatitis C necessary? Modelling the risk of removing hepatitis C antibody testing for Australian blood donations
Source: Vox Sang. 2023 May 14;118(6):480–7. doi: 10.1111/vox.13430 (PMC10952898; doi:10.1111/vox.13430)
Supplement: Supplementary file 1 — Data S1. Supporting information [file VOX-118-480-s001.docx]

**Supplementary Information for Paper**

**Testing strategy 1 further details methodology**

The baseline residual risks are as routinely calculated by Australian Red Cross Lifeblood and then a mean was used for the three periods.

Table1: Residual risks as calculated and averaged

| Period | Weusten Residual risk |
| --- | --- |
| 2015-2016 | 1 in 165 million |
| 2017-2018 | 1 in 96.6 million |
| 2019-2020 | 1 in 289.3 million |
| Mean | 1 in 151 million |

The Weusten model [1] finds the combined probability that virus is not detected during the window period AND that an infection develops in the recipient of the contaminated blood product.

This model assumes that the probability of infection depends linearly on the logarithm of the administered dose. The model also assumes that the 50% infectious dose is 10 copies and that the viral doubling time is 0.74 days. Finally, the model assumes detectability of low copy numbers of viral DNA or RNA by the screening assay can be described with a probit model [2]. Three periods were used 2015-2016, 2017-2018 and 2019-2020 and the mean of the three periods was the calculated baseline residual risk.

**Testing strategy 3 further details methodology**

Prior to 2017, Lifeblood annually estimated this probability for fresh components using the classical Incidence-WP (I-WP) model [3]. This widely applied deterministic model assumes that the residual risk for HIV, HCV and HBV is almost completely due to the WP risk – i.e. that the risk from other sources including viral variants and quarantine errors is so low that it can be ignored[4]. The I-WP model uses donation test results to determine the incidence rate (I) - the rate at which repeat donors acquire new infection (i.e. a positive donor with a negative result for the same virus within the past 12 months) per unit of time (person years of observation – the duration of time between negative tests for all donors tested). The incidence rate is then multiplied by the annualised risk of an infected donor attending during the WP (i.e. the WP in days divided by 365). Thus, the residual risk is directly proportional to the incidence rate and the WP duration.

P = I x WP

The model output is the probability of collecting a donation from a donor in the WP, and so is applicable to estimate the risk of collecting an HCV window period contaminated plasma for fractionation unit that is the baseline residual risk. Note that this is different to the baseline residual risk of fresh components as it does not include a transmission factor built in and is simply the risk of collecting a window period donation. (Table 1). Lifeblood NAT and serological test results were used to identify incident plasma for fractionation donors. To calculate the baseline HCV residual risk is combined 2019 and 2020 data was used since there were no incident plasma for fractionation donors recorded in 2020. As Lifeblood has implemented mini-pool NAT (pools up to 16 plasma donations) using the Grifols Ultrio Elite assay, the WP estimates were adjusted accordingly. WPs specific to Ultrio Elite in MP16 were taken from Galel *et al*. [1] (WP plausible ranges derived by Lifeblood).

**Table 2: Baseline HIV, HCV and HBV WP residual risk estimates for plasma for fractionation donations**

| Marker | Plasma donations | Incident donors | Infectious  WP (MP16) | Person-Years (PY) | Incidence  (per 100,000 PY) | Residual risk (plausible range) | Predicted WP donations issued annually |
| --- | --- | --- | --- | --- | --- | --- | --- |
| HCV | 1,483,366 | 1 | 4.3 (4.0–4.8) days | 331,107 | 0.30 | 1 in 28.1(25.1–30.2) million | 0.027 |

### Maximum viral load in a contaminated donation

The maximum possible undetected viral load for a tested pool can be predicted by multiplying the 95% limit of detection (LOD) for the assay by the pool size. The analytical sensitivity of NAT testing in ID-NAT compared to MP-16 is outlined in Table 4.

**Table 3 – Predicted maximum undetected viral load in ID-NAT and MP-16 for the Ultrio Elite assay**

| Pool size | Viral load IU/mL |
| --- | --- |
|  | HCV |
| ID-NAT* | 3.0 (2.5–3.9) |
| MP-16 | 48.0 (40.0–62.4) |

* 95% LOD from manufacturer’s Australian package insert

1 Galel SA, Simon TL, Williamson PC, AuBuchon JP, Waxman DA, Erickson Y, et al. Sensitivity and specificity of a new automated system for the detection of hepatitis B virus, hepatitis C virus, and human immunodeficiency virus nucleic acid in blood and plasma donations. Transfusion. 2018; 58: 649-59.

2 Bliss CI. The Method of Probits. Science. 1934; 79: 38-9.

3 Seed CR, Kiely P, Keller AJ. Residual risk of transfusion transmitted human immunodeficiency virus, hepatitis B virus, hepatitis C virus and human T lymphotrophic virus. Intern Med J. 2005; 35: 592-8.

4 Glynn SA, Kleinman SH, Wright DJ, Busch MP. International application of the incidence rate/window period model. Transfusion. 2002; 42: 966-72.
